# Different Emergency Response Strategies to Oil Spills in Rivers Lead to Divergent Contamination Compositions and Microbial Community Response Characteristics

**DOI:** 10.3390/microorganisms13061193

**Published:** 2025-05-23

**Authors:** Xinyu Wen, An Fan, Jinsong Wang, Yulin Xia, Sili Chen, Yuyin Yang

**Affiliations:** 1 School of Civil Engineering, University of South China, Hengyang 421001, China; 2 South China Institute of Environmental Sciences (SCIES), Ministry of Ecology and Environment (MEE), Guangzhou 510655, China

**Keywords:** oil spill, emergency response, washing oil, microbial community, river sediment

## Abstract

Oil spills in inland rivers pose a significant threat to the surrounding environment, and the emergency response differs greatly from that in ocean or coastal areas. In this study, we focused on several commonly used emergency water treatment strategies in China’s inland oil spills, as well as the spilled washing oil in a serious accident case. We investigated the changes in oil-related chemical components before and after water treatment using GCxGC-TOF MS (Comprehensive Two-dimensional Gas Chromatography Time of Flight Mass Spectrometer). We tracked the shifts of microbial communities in the microcosms incubated with clean river water, simulated oil-contaminated water, and the treatment effluent. The results revealed that typical components, especially nitrogen-containing heterocyclic compounds, had different removal efficiencies among treatments. The diversity, composition, and potential functions of microbial communities responded differently to the treatments, and could be related to various substances, including PAHs (polycyclic aromatic hydrocarbons) and heterocyclic compounds. A few genera, such as *SC-I-84*, exhibited a high correlation with washing oil-related components and could serve as an indicator in such an oil spill emergency response. Our findings indicated that simply using petroleum oil or PAHs to evaluate oil spills was likely to underestimate the ecological impact, especially when the spilled substances were coal chemical products widely used in China. This will provide an important scientific basis for decision-making and strategy evaluation in emergency responses to inland oil spills.

## 1. Introduction

Oil spills are a global issue that pose a significant threat to the economy, environment, and ecosystem [1,2,3]. Although most of the studies focused on accidents in the ocean or coastal areas, oil spills in inland rivers also happen frequently and are attracting increasing attention [4,5,6]. Due to the weak dilution effect and close distance to residues, oil spills in inland rivers tend to have an urgent impact on the environment and society [7,8]. Moreover, the proximity of rivers to human settlements amplifies public concern due to perceptible sensory alterations caused by oil spills, such as odor emissions and visible discoloration. Crucially, as rivers often serve as primary potable water sources for adjacent communities, oil contamination directly jeopardizes drinking water safety, posing immediate health risks [9,10]. The treatment of oil-contaminated water is challenging and may lead to water supply interruptions, causing substantial economic losses and severe social impacts [5]. Therefore, various emergency response approaches have been adopted to mitigate the ecological and environmental impact of oil spill accidents, with parallel implementation to curb associated economic losses.

In coastal oil spill accidents, in situ burning, mechanical recovery, and oil spill dispersants are widely used [11,12,13]. In contrast, in rivers, due to the sensitivity of freshwater aquatic organisms to the oil, more effective techniques are needed to further reduce the contamination. In situ and semi-in situ approaches, such as aeration, adsorption, and coagulation, are used more frequently [14,15,16,17]. Usually, oil-contaminated water is intercepted or controlled within a small area, such as the upstream of dams or ponds near the river. After most of the floating and dissolved oil has been removed, the water can be discharged back into the river.

However, significant challenges lie in evaluating the effectiveness and feasibility of these emergency response approaches. The pollutants referred to by the phrase ‘oil’ cover a variety of products with significant differences in composition and physicochemical characteristics [18,19], leading to great difficulty in describing the contamination levels with a single index. In practice, TPHs (total petroleum hydrocarbons) or petroleum oils could be used to comprehensively reflect the concentration of pollutants [20,21]. However, the various substances they refer to are usually reduced inconsistently during water treatment. For example, the removal rate of cycloalkanoic acids with a high ring number (Ox-NAs) was several times higher than that of cycloalkanoic acids with a low ring number (O2-NAs) when coagulation and adsorption were adopted to treat oily industrial wastewater [22]. The differences in biological toxicity of these components make the situation more complex: O2-NAs, which were harder to remove from water, were also more toxic to organisms, resulting in a high biological toxicity of the effluent [22]. Therefore, when the removal rate of a single indicator is used to describe the effectiveness of water treatment, it could always lead to underestimation or overestimation of the ecotoxicity and the subsequent ecological impact.

The impact of complex components has attracted much attention in the study of marine oil spills [13,23,24]. Various types of oil products differed greatly in biological toxicity due to their diverse chemical compositions [23,25]. They also performed differently when exposed to emergency response strategies such as oil dispersant [13,26]. It has been found since 2005 that the increased toxicity of the dispersed oil was related to the increased concentration of high molecular weight PAHs (polycyclic aromatic hydrocarbons) in the dissolved components [27]. Up to now, research has mainly focused on the PAH components in petroleum products in the ocean [24,28]. Knowledge was very limited about the chemical components of oil-like products from the coal chemical industry, which often involve highly toxic compounds such as heterocyclic compounds [29].

In contrast to the petroleum-centric chemical industry structure predominant in Europe and America, China’s coal chemical industry represents a highly developed sector with distinct technological pathways [30]. The extensive storage, transportation, and utilization of coal-derived products (e.g., washing oil) introduce environmental risks that lack established mitigation counterparts in the international domain. This study provides critical technical references for emergency response strategies targeting coal-derived oil-like product spill incidents, addressing a technological void in global environmental protection frameworks.

In 2022, an accidental oil pollution incident occurred in Guizhou Province, China. Washing oil, one of the fractions of coal tar, as well as a common material in the coal chemical industry, leaked into the Xiaohuangni River. The local government intercepted the river, introduced the polluted water to a nearby pond, and adopted various water treatment strategies to reduce the contamination before discharging the water downstream of the river. China’s strict water standards for petroleum oil (0.05 mg/L) restricted the application of biotechnology in emergency responses, so only physical/chemical water treatment methods were adapted. Over 80% of the contaminants were believed to be removed, as evaluated according to the level of petroleum oil. However, little was known about the chemical composition and ecological toxicity (or potential impact) of the effluent.

The current study revisits the washing oil pollution incident in Xiaohuangni River, the accident with the longest emergency response duration in China. The objects of this study are to (1) systematically demonstrate the changes in chemical components of washing oil caused by different water treatment processes; (2) unveil the structural and potential functional shift of microbial community exposed to the different treated oil-contaminated water; (3) explore the correlation between community response characteristics and the chemical composition. The results will help evaluate the effectiveness of emergency measures from a microbial ecological perspective and provide basic information for long-term bioremediation in the future.

## 2. Materials and Methods

### 2.1. Sample Collection

Water and sediment samples used to set up the microcosms were collected from Xiaohuangni River, which encountered an accidental washing oil pollution event in 2022 [31]. The sampling site was located at 25°24′53.74″ N, 104°37′40.13″ E, approximately 1 km upstream of the pollution site. The water depth was approximately 2 m at the site. The sampling site is located in Panzhou City, Guizhou Province, and the temperature is usually maintained at 20–30 °C.

Surface sediment (0–10 cm) was collected using a grab sampler. The corresponding overlying water was collected using a plexiglass water sampler. The temperature, dissolved oxygen, pH, and conductivity of the samples were measured immediately at the sampling site using portable instruments. All of the samples were stored in HDPE containers and transported to the laboratory at 4 °C.

### 2.2. Simulation of Water Treatment Process

The contaminated water was simulated by adding 120 mg of washing oil to 6 L of uncontaminated river water. Tween 80 was added to the mixture at a final concentration of 5 ppm, and the mixture was stirred for 30 min to ensure the emulsification of the washing oil. The mechanical dispersion method was employed for mixing, which effectively simulates natural mixing characteristics observed in environmental conditions [32]. The concentration of washing oil is 20 mg/L, close to that at the initial stage of the sudden pollution of the Xiaohuangni River. The 20 mg/L oil-containing water sample was subjected to four different water treatment techniques as follows:

Adsorption: Adding 20 mg of powdered activated carbon to 1 L of oil-contaminated water, stirring at 150 rpm for 30 min, and then filtering using a diaphragm vacuum pump.

Aeration: Aerate the oil-contaminated water for 6 h at a gas-to-water ratio of 20:1.

Coagulation: Add 0.2 g/L of polyacrylamide (PAM), 0.9 g/L of polyaluminum chloride (PAC), and 1 mg/L of sodium hydroxide to the oil-contaminated water. Stir at 300 rpm for 2 min, 100 rpm for 2 min, 70 rpm for 1 min, and let it stand for 30 min before siphoning the supernatant.

Demulsification–coagulation: Add potassium ferrate to the contaminated water at a final concentration of 3 g/L, stir at 800 rpm for 30 secs, 400 rpm for 5 min, 100 rpm for 10 min, and then let it stand for 5 h. After demulsification, the coagulation process is the same as mentioned above.

The treated water samples were collected in brown glass bottles, stored at 4 °C, and subjected to chemical analysis and microcosm setup within 24 h.

### 2.3. Chemical Analysis

The physicochemical parameters of the environmental samples, including TN (total nitrogen), ammoniacal nitrogen, nitrate, nitrite, and TP (total phosphate), were determined following the national environmental standard methods as required by the Environmental Protection Agency of China (2002).

The chemical composition of the organic contaminants before and after the treatment process was analyzed using GCxGC-TOF MS (GGT 0620, Hexin Instruments, Guangzhou, China). The oily water sample was added with sodium chloride, stirred and dissolved, then transferred to a separating funnel and extracted with 30 mL of dichloromethane. The extraction was repeated three times, and the extracted solutions were combined and concentrated under vacuum at 40 °C, then diluted with dichloromethane to 1 mL for machine detection. In the comprehensive two-dimensional gas chromatography, the two gas chromatography columns used were DB-5MS (30 m × 0.25 mm × 0.25 μm) and DB-17 (1.3 m × 0.18 mm × 0.18 μm), respectively. An HV modulation column was used, and the modulation period was set to 4 s. The column temperature was kept at 60 °C for 3 min, raised to 315 °C at the rate of 6 °C/min, and maintained for 15 min. Ions with a mass in the range of 45–500 amu were collected by mass spectrometry at a collection rate of 101 spectra per second.

### 2.4. Microcosm Setup

Surface sediments are critical zones for material cycling and energy metabolism in rivers, with active biological activity [33]. Compared to flowing river water, pollutants entering sediments are less susceptible to dilution and natural cleansing, exerting ecological impacts over longer timescales [34,35]. In addition, washing oil exhibits a density of 1.03–1.06 g/cm^3^ (higher than that of water) at ambient temperature [36], whereas most crude oils range between 0.746 and 1.016 g/cm^3^ [21]. This means that the wash oil is more likely to enter the water and contact the sediment. Therefore, we focused on microbial community responses in sediments.

Microcosm incubation was carried out in 500 mL conical flasks containing 100 g of sediment and 160 mL of water with/without oil contamination. A total of six groups were set up. In the BLK group, the original river water was used as an uncontaminated control. In the POL group, untreated oil-contaminated water was added to simulate the effect of an oil spill without emergency response. In the ADT, AET, COT, and DCT groups, contaminated water treated by adsorption, aeration, coagulation, and demulsification–coagulation was used, respectively. Three biological replicates were set up for each treatment.

The flasks were shaken vigorously to mix the sample thoroughly, and then incubated at 25 °C (close to the in situ temperature at the time of sampling) and 100 rpm. Approximately 2 mL of slurry samples were collected from each flask on days 0, 3, 7, 14, 21, and 28 to evaluate the shift of the microbial community. The slurry samples were centrifuged at 4 °C and 12,000 rpm for 1 min and kept at −20 °C after discharging the supernatant.

### 2.5. Amplicon Sequencing

Genomic DNA was extracted from the slurry samples using a TIANamp Soil DNA Kit (Tiangen Biotech; Beijing, China) following the manufacturer’s instructions. After examining the quality of the nucleic acid using agarose gel electrophoresis, samples were subjected to amplicon sequencing on an Illumina MiSeq platform. The primer sets 515F/907R (5′-GTGCCAGCMGCCGCGGTAA-3′/5′-CCGTCAATTCMTTTRAGTTT-3′) targeted the V4-V5 region of bacterial 16S rRNA gene were adopted to evaluate the microbial diversity [37]. Raw sequence data were deposited in the NCBI Sequence Read Archive (SRA) under the project number PRJNA1226169.

### 2.6. Data Analysis and Visualization

Sequencing data were processed following the Qiime2 pipeline [38]. Raw sequences were demultiplexed and quality trimmed, and the amplicon sequence variants (ASVs) and a feature table were obtained from the chimer-free sequences with DADA2 [39]. The feature table was subsampled to the minimum number of sequences (approximately 6000) to obtain comparable diversity information. Then, the alpha and beta diversity metrics were calculated accordingly. Taxonomy annotation was carried out using a Bayesian classifier trained with sequences from the Silva release 128 [40]. Functional prediction was performed with PICRUSt2 [41], based on the feature table and the corresponding phylogenetic tree constructed using FastTree (2.1.11) [42]. The predicted community function was collapsed into KOs (KEGG Orthologies) and KEGG pathways based on the KEGG database [43].

Further data processing and analysis were carried out in R software (4.4.1, R Core Team, 2024) with packages tidyverse (2.0.0) [44] for general data processing, and vegan (2.6.6.1) [45] for numerical ecology analysis and statistical tests. PERMANOVA (permutational multivariate analysis of variance) was applied to examine the statistical difference among microbial communities [46]. The *p*-value was calculated based on 999 random permutations. Visualization was performed with R packages ggplot2 (3.5.1) [47], pheatmap (1.0.12) [48], and ggrepel (0.9.6) [49].

## 3. Results

### 3.1. Oil-Related Chemical Composition After Treatment

**Figure 1:**
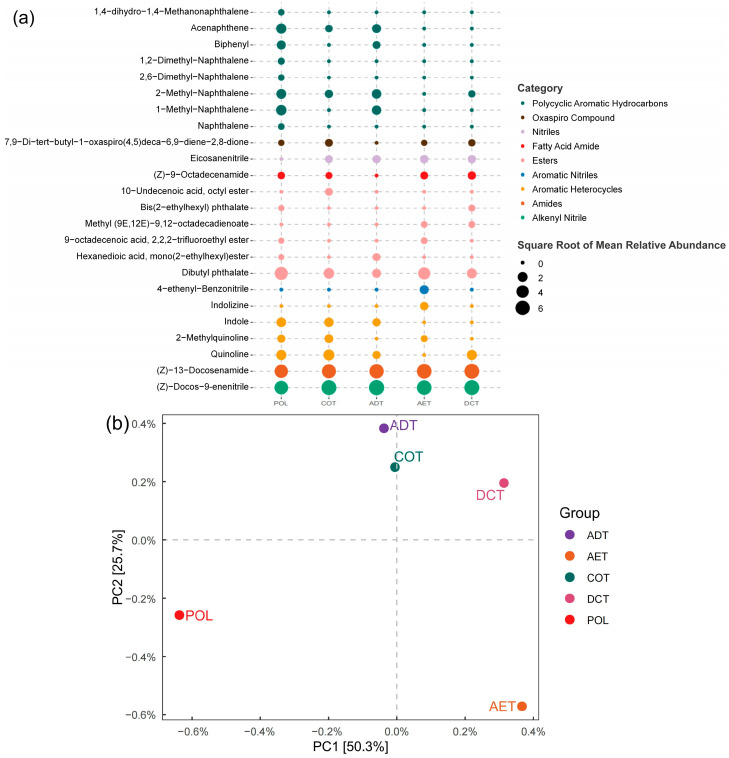
The composition of washing oil-related semi-volatile organic compounds before and after water treatment. (**a**) heatmap showing the compounds with an average relative abundance of over 0.15%. (**b**) PCA giving an overview of organic chemical composition of each sample.

Various kinds of sVOCs (semi-volatile organic compounds) were detected in the washing oil-containing water, and a notable difference could be observed in the chemical compositions of the treated effluent (Figure 1a). The most abundant substances in the POL group, including (Z)-docos-9-enenitrile (26.19%), (Z)-13-docosenamide (24.69%), and dibutyl phthalate (21.62%), decreased remarkably after treatment. PAHs (polycyclic aromatic hydrocarbons) were easily reduced by all the treatments. Biphenyl and 1-methyl-naphthalene, for example, became undetectable in the AET, COT, and DCT effluent. On the other hand, some of the compounds seemed to be reduced only by certain processes, resulting in different composition patterns in the effluent. Nitrogenous heterocyclic compounds accounted for 9.55% of the peak area of the detectable sVOCs in the untreated water (POL), but the percentage varied from 1.60% to 12.81% in the treated water. Indole and quinoline decreased remarkably after aeration (AET), while they remained unaffected by coagulation (COT).

PCA (principal component analysis) was adopted to reduce the dimensionality of the above-mentioned chemical composition data (Figure 1b). The results showed that the water treatment processes changed the chemical composition of the oil-contaminated water remarkably. Among the various treatment approaches, adsorption, coagulation, and demulsification–coagulation affected the chemical components in a similar way, while aeration led to a much different pattern of sVOC characteristics of the effluent.

### 3.2. Microbial Diversity Shifts During the Incubation

**Figure 2:**
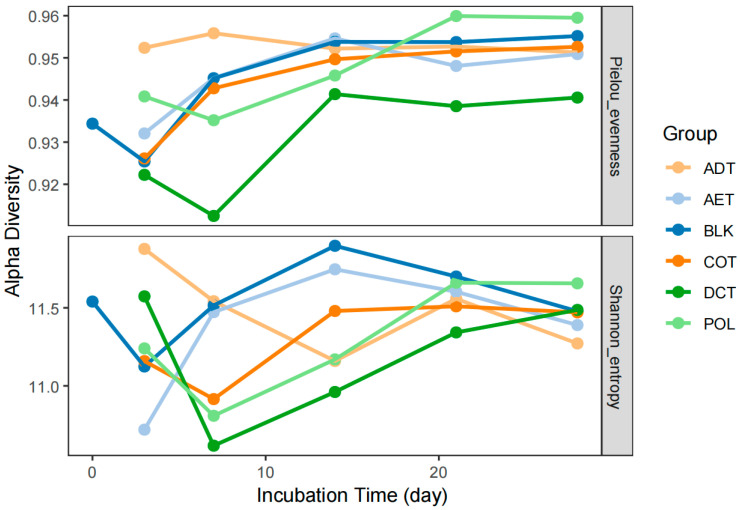
Variation in microbial alpha diversity in each microcosm incubated with sediment and simulated contaminated water with/without treatment.

During the 4-week incubation, Pielou’s evenness and Shannon diversity index of the bacterial community in sediment fluctuated slightly between 0.91~0.96 and 10.62~11.90, respectively (Figure 2). Notable differences in variation trend could be observed not only between blank control (BLK) and contamination control (POL), but also among different treatments. After exposure to washing oil contaminated water (POL), the Pielou’s evenness index of microbial community in river sediment encountered a decrease in the first week, but then increased and exceeded the others. Treated groups AET and COT had Pielou’s evenness indices close to that of the blank group (BLK). Microcosms incubated with demulsification–coagulation treated water (DCT) had the lowest evenness. The group ADT maintained a high and stable level of sedimentary microbial richness during the incubation period.

In most of the microcosms, the Shannon index decreased in the first few days and then rose again. However, the Shannon index in microcosm ADT continued to decline in the first two weeks and remained at a lower level in the following two weeks. The level and trend of the Shannon diversity in the AET microcosm were very similar to those in BLK. At the same time, the groups COT and DCT underwent a similar variation process with POL.

**Figure 3:**
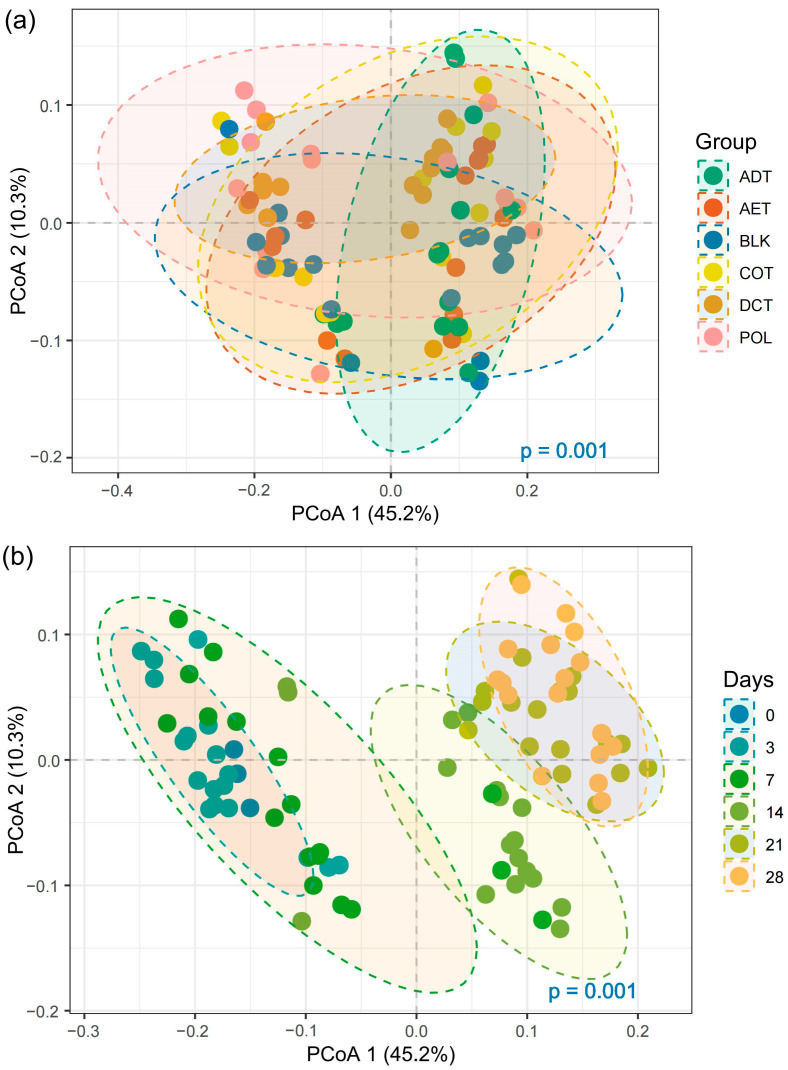
PCoA of microbial community structure based on weighed UniFrac distance, focusing on (**a**) treatment, and (**b**) temporal variation. Treatments ADT, AET COT and DCT refer to adsorption, aeration, coagulation and demulsification–coagulation, respectively. While treatment POL refers to untreated simulated oil-contaminated water, and BLK refers to blank control.

Weighted-UniFrac distance-based PCoA (Principal Coordinate Analysis) gave an overview of the beta-diversity of microbial communities in the microcosms, as shown in Figure 3. PERMANOVA result indicated a significant shift among different treatments (Figure 3a, *p* < 0.001). As shown by the 95% confidence ellipses, AET and COT microcosms shared a similar community structure. In contrast, the microbial community in the ADT microcosm showed a greater dissimilarity with those of other treatments. The microbial community also varied notably with incubation time (Figure 3b). Variation was insignificant in the first week. But after 2 weeks’ incubation, microbial community structure had a great shift in all the microcosms. The communities tended to be stable after the third week.

### 3.3. Dynamics of Microbial Composition

**Figure 4:**
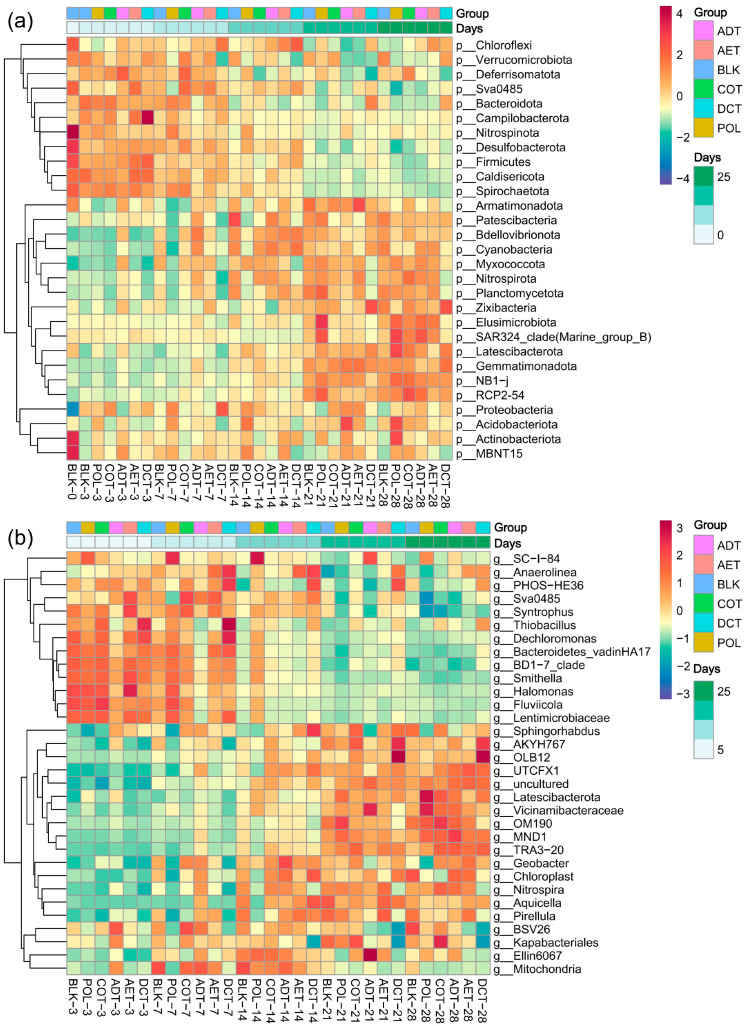
Heatmap of dominant microbial groups during incubation and among treatments at (**a**) phylum level, and (**b**) genus level. Treatments ADT, AET COT and DCT refer to adsorption, aeration, coagulation and demulsification–coagulation, respectively. While treatment POL refers to untreated simulated oil-contaminated water, and BLK refers to blank control.

A total of 69 phyla were detected in the sediment samples, 23 of which had an average relative abundance of over 1% (Figure 4a). Proteobacteria (29.91%), Bacteroidota (16.37%), and Chloroflexi (11.20%) were among the most abundant phyla in the uncontaminated control (BLK) and were affected in all the treatments. More subtle changes in community structure can be observed at the genus level. At the initial stage of incubation (3–7 days), the genus-level composition in the adsorption microcosm seemed different from that of the other treatments. Later, during 3–4 weeks, the dominant genera in the demulsification–coagulation microcosm diverged from others more clearly.

Some of the dominant genera varied more notably between treatments. In the microcosm exposed to untreated oil-containing water, the genus *SC-I-84* had a remarkably high relative abundance of 1.06% on average, higher than that of the blank control and other treatments during the incubation. It was significantly enriched during incubation, peaked on day 14 (1.53%), and then began to decline. *Thiobacillus* sp. varied obviously during the first week, enriched in ADT but declined in DCT. In contrast, BSV26 and *Nitrospira* sp. increased in ADT but decreased in DCT, and were particularly evident in the later stages of incubation.

### 3.4. Functional Shifts of Microbial Communities

**Figure 5:**
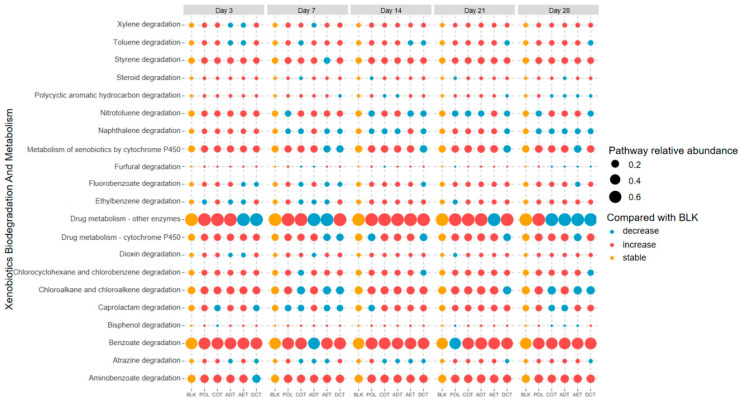
Shift of xenobiotic degradation function in each sample compared with the blank control. Treatments ADT, AET COT and DCT refer to adsorption, aeration, coagulation and demulsification–coagulation, respectively. Treatment POL refers to microcosm with untreated simulated oil-contaminated water, and BLK refers to blank.

**Figure 6:**
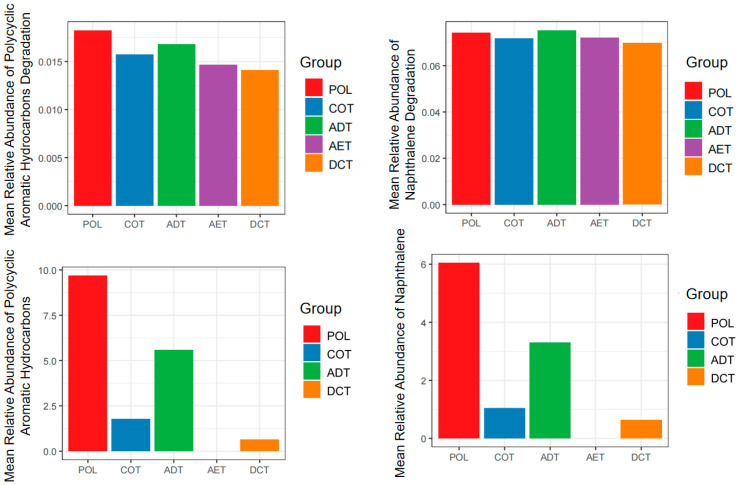
The levels of PAHs and naphthalene composition and corresponding predicted function abundance on day 3. Treatments ADT, AET COT and DCT refer to adsorption, aeration, coagulation and demulsification–coagulation, respectively. Treatment POL refers to microcosm with untreated simulated oil-contaminated water, and BLK refers to blank.

Function prediction was carried out based on the sequence data, and emphasis was put on the xenobiotic degradation pathways according to the KEGG (Kyoto Encyclopedia of Genes and Genomes) database. We compared the predicted function abundance of each treatment with the blank control to examine whether they might be enriched during incubation (Figure 5). At the beginning of the incubation (days 3–7), most of the xenobiotic degradation functions were enriched, especially PAHs degradation (M00626) and naphthalene degradation (M00624), indicating a functional adaptation to the oil-related contamination. With a prolonged incubation time, a higher proportion of pathways decreased compared with the blank control. We compared the total peak area percentage of the PAHs in contaminated water added to the microcosms with the predicted degradation pathway abundance of PAHs, as well as those for naphthene (Figure 6). Except for the aeration treatment (AET) in which PAHs were completely removed, a positive rank correlation between the upregulation of functions and the proportion of residual PAHs (or naphthalene) could be observed.

Differences also existed among treatments. In the microcosm incubated with untreated oil-containing water (POL), most of the functional pathways had a higher relative abundance. The coagulation-treated microcosm (COT) shared a similar pattern to that of POL. But many of the function pathways related to aromatic compound degradation decreased in the aeration (AET) and demulsification–coagulation (DCT) microcosms.

## 4. Discussion

### 4.1. Chemical Characteristics of Pollution

The chemical composition of oils was considered to be the key to their environmental behavior, fate, and toxicity, and therefore made great sense in emergency response decision making [23,25]. As suggested by CROSERF (Chemical Response to Oil Spills Ecological Effects Research Forum), the chemical composition, especially a detailed hydrocarbon compound composition of the oil product, should be described to acquire comparable research results [50]. However, only 5% of the studies followed the guidance as revealed by a subsequent meta-study, leading to confusing and inconsistent results in most oil toxicity studies [51].

The result of the current study further showed that hydrocarbon compounds only accounted for a small portion of the washing oil, approximately 12.02% of the sVOC peak area. GCxGC-TOF MS was adopted in the study to provide non-targeted chemical composition data, in order to cover as many potential toxic components as possible. The method had been proven highly effective in the chemical composition analysis of oils, with the ability to separate up to 15,000 different components [52]. It could also be used to provide pollution fingerprints of different oil products in oil spills [53]. In the case we studied, the spilled product, washing oil, was a fraction obtained by distilling coal tar at 230–300 °C, accounting for approximately 4.5–10% of the coal tar [36]. The relatively simple chemical composition (compared to coal tar) helped to compare composition shifts in the water treatment process.

A total of 28 chemicals from 12 categories were identified. As shown in Figure 1a, PAHs, which attracted much attention in previous studies [54,55,56], only accounted for 17.01% of the peak area in the washing oil-contaminated water. While others, such as esters (21.90%) and heterocyclic compounds (26.56%), which had long been ignored, also comprised a considerable proportion of the oil-related organic compounds. Due to the quantitative limitations of the non-targeted analysis, the peak area cannot accurately reflect the actual concentration [57,58]. The results indicated that focusing solely on hydrocarbons in oil component analysis could lead to an inappropriate underestimation of their environmental impact.

Nitrogen-containing heterocyclic compounds, such as quinoline, were also harmful to aquatic and terrestrial organisms. For example, quinoline had a similar acute oral toxicity to rats as naphthalene [59,60]. Although most of the heterocyclic compounds identified in washing oil had a lower toxicity to aquatic organisms compared with typical PAHs such as naphthalene, at least two factors indicated that they deserved more attention: Firstly, due to the increased polarity of molecules caused by nitrogen, heterocyclic compounds such as quinoline usually had a stronger water solubility than PAHs [61]. This meant that in the event of a sudden and severe oil spill, heterocyclic compounds would present a far higher concentration in the aqueous phase, causing possible damage to aquatic organisms. Secondly, as revealed by the current study (Figure 1a), PAHs were easily removed in common emergency water treatment processes such as coagulation and aeration, while heterocyclic compounds were not. Previous studies on industrial coking wastewater treatment had also reported similar failures in quinoline removal [62,63]. More importantly, the water treatment approach and the type of heterocyclic compounds seemed to have an interactive effect on the removal rate, implying that detailed chemical analysis before and after water treatment was crucial for evaluating the effectiveness of emergency response.

Due to the coal-based energy structure, coal chemical raw materials, including coal tar and washing oil, were widely used in China [64,65]. Coal tar, for example, is a coal processing product characterized by complex compositions, serving as a source of diverse chemical compounds [66,67]. Therefore, more complex components and a higher content of heterocyclic compounds would enter the aquatic environment when these products were released. In addition, the method of determination of petroleum concentration was not suitable for the determination of the concentration of washing oil (Table S1 and Figure S1). Our study suggested that using a single indicator for petroleum oils in such events was likely to lead to limited or even erroneous insights in emergency response.

### 4.2. Microbial Community Shifts in Response to Washing Oil Contamination

Since the catastrophic blowout of Deepwater Horizon, a considerable amount of research has focused on the response of microbial communities to oil spills under on-site or laboratory conditions [68,69,70,71]. Under environmental stress, the reduction in sensitive species usually leads to a decrease in the richness and evenness of local microbial communities [71,72,73]. In the current study, we also observed a similar trend; for example, a decrease in Shannon diversity and Pielou’s evenness could be noticed in the first week of incubation in the POL microcosm (Figure 2). However, during the four-week incubation, the microbial community diversity of all the treatments was restored to varying extents. This may be related to the pollution levels set in the study: (1) washing oil contaminated river water, instead of a large amount of oil, was added to the microcosms; and (2) emergency response removed approximately 2.38–73.52% of pollutants from the water (in petroleum oil, measured according to HJ 970—2018, in Table S2). In addition, the concentration of pollutants such as PAHs would also decrease over time [74,75]. The degradation and adaptation of microorganisms to the oil-related organic components [76,77] might contribute to the restoration of the community.

A wide range of microbial groups, such as *Pseudomonas* sp., were reported to be enriched after an oil spill or addition, due to their ability to degrade petroleum or aromatic hydrocarbons [78]. The polycyclic aromatic hydrocarbons (PAHs) degradation capacity of *Pseudomonas* may primarily originate from the *ndo* gene encoding naphthalene dioxygenase identified in its genome [79]. Similar enrichment of *Dechloromonas* sp., which was among the well-known hydrocarbon degraders [80], was also confirmed in this study (Figure 4b). The relative abundance of *Dechloromonas* sp. had a clear variation over time, reflecting the selective effect of washing oil contamination on the microbial community. In addition, we noticed a remarkable increase in some previously unreported microbial genera in wash oil-contaminated systems. *SC-I-84*, for example, experienced a great increase in the POL microcosm, from 1.16% on day 3 to 1.53% on day 14 (Figure 4b). The response of genus SC-I-84 to environmental stress has been reported in many studies [81,82,83], but the lack of pure cultivation has limited the understanding of its functional characteristics. Recently, Li et al. systematically reported the metabolic function of SC-I-84 for the first time based on metagenome-assembled genomes from a bioreactor [84]. SC-I-84 was identified as a partially nitrifying bacterium, which carried nitrate reduction genes such as napA, napB, narY, and narV [84]. A complete gene set related to the TCA cycle was also present in its genetic map, indicating the potential for utilizing carbon sources [84]. Genomic evidence also suggested that SC-I-84 could produce trehalose that helped to resist harmful environments [84], which might explain its competitive advantage in polluted environments. Therefore, the remarkable enrichment of SC-I-84 in the POL group could be related to its tolerance to contamination. And its carbon metabolism and denitrification functions would contribute to the degradation of contaminants related to washing oil.

For a long time, PAHs were considered the main characteristic components causing biotoxicity in oils, and they had a persistent impact on microorganisms [85,86]. However, there was a weak positive correlation between the total peak area of PAHs and the microbial richness in early incubation, as indicated by Spearman’s correlation test (ρ = 0.70, *p* = 0.19). This can be explained by the effective removal of PAHs in the emergency response process. At lower concentrations, PAHs can be degraded by microorganisms and provided with energy and carbon sources [87]. On the other hand, the impact of other compounds, especially those with different removal efficiencies among treatments, might be underestimated in previous studies. Quinoline, a nitrogen-containing heterocyclic compound, had good water solubility and antibacterial effects [88,89] and was found to be weakly negatively correlated to microbial richness in early incubation (ρ = −0.23, *p* = 0.71). 7,9-Di-tert-butyl-1-oxaspiro (4,5) deca-6,9-diene-2,8-dione, a genotoxic carcinogen which also inhibits enzyme activity [90,91], had a similar negative correlation with Pielou’s richness index (ρ = −0.68, *p* = 0.01). The limited historical attention given to these compounds in oil spill incidents may have stemmed not only from the inherent variability in oil composition across different oil types but also from the heterogeneity of their interactions with native microbial communities. Consequently, the novel findings from this study are primarily oriented toward the remediation scenarios for wash oil contamination. These compounds warrant systematic investigation in future studies, as they may provide critical insights for developing targeted emergency mitigation strategies against coal chemical-related contamination events.

In general, the shift of microbial community structure may be related to various chemical substances in the microcosms. In some studies, ordination analysis methods such as CA (Correspondence Analysis) and RDA (Redundancy Analysis) were adopted to explore the relationship between environmental variables and community composition [92,93]. However, in our study, it was difficult to establish a reliable constrained model with limited samples due to the excessive number of constraint variables (oil-related chemical components) and the interaction between treatment and incubation time [94,95]. Therefore, we only focus on a few key microbial genera and toxic components, and leave the exploration of the numerical correlation between organic components and community structure to future exploration.

### 4.3. Potential Functional Shifts in Response to Washing Oil Contamination

Overall, microbial community functions might be damaged or adapted under pollution pressure, partly depending on the type, level, and duration of contamination [96,97,98]. Previous studies reported the upregulation of petroleum or aromatic hydrocarbon degradation function in in situ microbial communities after various oil spills [99,100], which was consistent with the findings of this study (Figure 5). A considerable number of xenobiotic degradation pathways underwent temporal changes, with the most obvious ones related to the degradation of PAHs and naphthalene. This also aligned well with the previously discussed stimulation of low concentrations of PAHs on degrading microorganisms [87].

However, in the aeration treatment (AET), although PAHs had been reduced to an undetectable level, the abundance of PAHs degradation pathways remained at a level second only to the pollution microcosm (POL). This might be related to the residue of some other aromatic substances in the effluent (Figure 1a), but further research is needed to confirm. Some of the organic components identified in oil-contaminated water are currently difficult to clearly associate with specific metabolic pathways and genes. It was still difficult to determine whether they caused more damage to microbial community function or stimulated degradation.

## 5. Conclusions

In this study, various chemical substances were identified from washing oil, a widely used coal tar fraction, using the untargeted GCxGC-TOF MS approach. The chemical composition of washing oil underwent remarkable changes during the simulated environmental emergency response process. And the removal efficiency of some toxic compositions, such as nitrogen-containing heterocyclic compounds, differed greatly among the treatments. In microcosms with simulated contaminated water and treated effluent, the structure and potential function of sediment microbial communities encountered shifts related to the composition of oil-related organic chemical compounds. Our results confirm that toxic components, such as nitrogen-containing heterocyclic compounds, were inappropriately underestimated in previous oil spill studies. The component-specific removal rates challenge the assumption of uniform efficacy in China’s traditional emergency response protocols. To prevent severe misjudgment of ecological risks, emergency decision-making for river oil spills should rely on whole-component removal rate assessment or ecotoxicity evaluation rather than single-indicator (such as petroleum oil) removal rate assessment.

## Data Availability

Raw sequence data were deposited in NCBI Sequence Read Archive (SRA) under the project number PRJNA1226169 at Available online: https://www.ncbi.nlm.nih.gov/sra/PRJNA1226169 (accessed on 20 April 2025).

## Supplementary Materials

The following supporting information can be downloaded at https://www.mdpi.com/article/10.3390/microorganisms13061193/s1, Figure S1: Standard curve based on oil determination method (HJ 970-2018); Table S1: The concentration of 10 mg/L water containing washing oil was determined according to the method of HJ 970-2018, and the obtained results were all different from 10 mg/L; Table S2: The absorbance of washing oil before and after water treatment was determined by HJ 970-2018. POL refers to blank control.

## Abbreviations

The following abbreviations are used in this manuscript:

PAHs	Polycyclic aromatic hydrocarbons
GCxGC-TOF MS	Comprehensive Two-dimensional Gas Chromatography Time of Flight Mass Spectrometer

## References

[1] Idomeh J., Shittu O., Oyedepo J., Bada B., Balogun S., Idomeh F., Ezenweani R. (2021). Petroleum Hydrocarbon Impacted Aquatic Ecosystem Reveals Methylotenera as the Dominant Genera in the Niger Delta Region of Nigeria. Geomicrobiol. J..

[2] Rezaei Somee M., Dastgheib S.M.M., Shavandi M., Ghanbari Maman L., Kavousi K., Amoozegar M.A., Mehrshad M. (2021). Distinct microbial community along the chronic oil pollution continuum of the Persian Gulf converge with oil spill accidents. Sci. Rep..

[3] Huettel M. (2022). Oil pollution of beaches. Curr. Opin. Chem. Eng..

[4] Lu J., Chen L., Xu D. (2024). Study on the Oil Spill Transport Behavior and Multifactorial Effects of the Lancang River Crossing Pipeline. Appl. Sci..

[5] Yang Y., Wang S., Zhu Z., Jin L. (2022). Prediction model and consequence analysis for riverine oil spills. Front. Environ. Sci..

[6] Adams J.E., Brown R.S., Hodson P.V. (2020). The bioavailability of oil droplets trapped in river gravel by hyporheic flows. Environ. Pollut..

[7] Chen H., Lu S., Shao Y., Wang B., Liu Y. (2023). Study on oil spill risk zoning in the Yangtze River Estuary based on the visited probability method of sensitive targets. J. Environ. Eng. Technol..

[8] Jiang P., Tong S., Wang Y., Xu G. (2021). Modelling the oil spill transport in inland waterways based on experimental study. Environ. Pollut..

[9] Lee H.-J., Kim H.-J., Yoo S.-H. (2018). The Public Value of Reducing the Incidence of Oil Spill Accidents in Korean Rivers. Sustainability.

[10] Cakmak S., Hebbern C., Cakmak J.D., Dales R.E. (2017). The influence of polycyclic aromatic hydrocarbons on lung function in a representative sample of the Canadian population. Environ. Pollut..

[11] Fritt-Rasmussen J., Wegeberg S., Lassen P., Wilms L.B., Renvald L., Larsen M.B., Geertz-Hansen O., Wiktor J., Gustavson K. (2023). Coastline in-situ burning of oil spills, analysis of a Greenland field experiment. J. Hazard. Mater..

[12] An W., Zhang Q., Zhao J., Qu L., Liu S., Yang M., Xu J. (2021). Mechanism Investigation on a Novel Oil Recovery Skimmer Coupling Free Surface Vortex and Cyclone Separation. ACS Omega.

[13] Adofo Y.K., Nyankson E., Agyei-Tuffour B. (2005). Dispersants as an oil spill clean-up technique in the marine environment: A review. Heliyon.

[14] Wan L., Liu Y., Yang Z. (2022). Preparation of oil-removal materials and their enhanced removal effects on oily substances in coal chemical wastewater. Water Treat. Technol..

[15] Vieira P.A., Vieira R.B., Faria S., Ribeiro E.J., Cardoso V.L. (2009). Biodegradation of diesel oil and gasoline contaminated effluent employing intermittent aeration. J. Hazard. Mater..

[16] Zhang Y. (2021). Research on the Methods and Effects of Oil Removal in Coal Chemical Wastewater. Master’s Thesis.

[17] Wu Q. (2018). Study on Adsorption-Coagulation Emergency Treatment of Petroleum Pollution in Drinking Water Sources. Master’s Thesis.

[18] Arey J.S., Martin Aparicio A., Vaiopoulou E., Forbes S., Lyon D. (2022). Modeling the GCxGC Elution Patterns of a Hydrocarbon Structure Library To Innovate Environmental Risk Assessments of Petroleum Substances. Environ. Sci. Technol..

[19] Beyer J., Trannum H.C., Bakke T., Hodson P.V., Collier T.K. (2016). Environmental effects of the Deepwater Horizon oil spill: A review. Mar. Pollut. Bull..

[20] Mirjani M., Soleimani M., Salari V. (2021). Toxicity assessment of total petroleum hydrocarbons in aquatic environments using the bioluminescent bacterium Aliivibrio fischeri. Ecotoxicol. Environ. Saf..

[21] Munnelly R.T., Windecker C.C., Reeves D.B., Rieucau G., Portier R.J., Chesney E.J. (2021). Effects of short-duration oil exposure on bay anchovy (*Anchoa mitchilli*) embryos and larvae: Mortality, malformation, and foraging. Aquat. Toxicol..

[22] Wang Q., Li Y., Zhang X., Wang X., Zhu S., Li Z., Chen C. (2023). Research progress of removal technologies of naphthenic acids in petroleum and petrochemical wastewaters. Ind. Water Treat..

[23] Philibert D.A., Lyons D.D., Tierney K.B. (2021). Comparing the effects of unconventional and conventional crude oil exposures on zebrafish and their progeny using behavioral and genetic markers. Sci. Total Environ..

[24] Ruberg E.J., Elliott J.E., Williams T.D. (2021). Review of petroleum toxicity and identifying common endpoints for future research on diluted bitumen toxicity in marine mammals. Ecotoxicology.

[25] Xin Q., Saborimanesh N., Ridenour C., Farooqi H. (2024). Fate, behaviour and microbial response of diluted bitumen and conventional crude spills in a simulated warm freshwater environment. Environ. Pollut..

[26] Shi D., Jia H. (2023). Transport and behavior of marine oil spill containing polycyclic aromatic hydrocarbons in mesocosm experiments. J. Oceanol. Limnol..

[27] Couillard C.M., Lee K., Légaré B., King T.L. (2005). Effect of dispersant on the composition of the water-accommodated fraction of crude oil and its toxicity to larval marine fish. Environ. Toxicol. Chem..

[28] Cao Y., Zhang B., Greer Charles W., Lee K., Cai Q., Song X., Tremblay J., Zhu Z., Dong G., Chen B. (2022). Metagenomic and Metatranscriptomic Responses of Chemical Dispersant Application during a Marine Dilbit Spill. Appl. Environ. Microbiol..

[29] Ma Z.-H., Wei X.-Y., Liu G.-H., Liu Z.-Q., Liu F.-J., Zong Z.-M. (2019). Insight into the Compositions of the Soluble/Insolube Portions from the Acid/Base Extraction of Five Fractions Distilled from a High Temperature Coal Tar. Energy Fuels.

[30] Shi J., Chunyan X., Yuxing H., Han H. (2021). Case study on wastewater treatment technology of coal chemical industry in China. Crit. Rev. Environ. Sci. Technol..

[31] Zhu B., Huang D., Xu L., Xie W., An S., Wang C., Gong X., Zhang Z., Li W., Zhu L. (2024). Simulation and Analysis of Oil Pollutant Diffusion in River Affected by Coking Wash Oil Contaminated Groundwater. Earth Environ. Sci..

[32] Wang C., Han L., Zhang Y., Jiang A., Wang J., Niu X. (2024). Effects of Physical Properties and Environmental Conditions on the Natural Dispersion of Oil. J. Mar. Sci. Eng..

[33] Zhao Z., Zhao R., Qiu X., Wan Y., Lee L. (2022). Structural Diversity of Bacterial Communities and Its Relation to Environmental Factors in the Surface Sediments from Main Stream of Qingshui River. Water.

[34] Yan Z., Hao Z., Wu H., Jiang H., Yang M., Wang C. (2019). Co-occurrence patterns of the microbial community in polycyclic aromatic hydrocarbon-contaminated riverine sediments. J. Hazard. Mater..

[35] Zhang W., Zhu M., Li Y., Wang C., Qian B., Niu L., Wang P., Gu J., Yang N. (2020). How fluvial inputs directly and indirectly affect the ecological status of different lake regions: A bio-assessment framework. J. Hydrol..

[36] (2009). Wash Oil.

[37] Angenent L.T., Kelley S.T., St Amand A., Pace N.R., Hernandez M.T. (2005). Molecular identification of potential pathogens in water and air of a hospital therapy pool. Proc. Natl. Acad. Sci. USA.

[38] Caporaso J.G., Kuczynski J., Stombaugh J., Bittinger K., Bushman F.D., Costello E.K., Fierer N., Peña A.G., Goodrich J.K., Gordon J.I. (2010). QIIME allows analysis of high-throughput community sequencing data. Nat. Methods.

[39] Callahan B.J., McMurdie P.J., Rosen M.J., Han A.W., Johnson A.J., Holmes S.P. (2016). DADA2: High-resolution sample inference from Illumina amplicon data. Nat. Methods.

[40] Quast C., Pruesse E., Yilmaz P., Gerken J., Schweer T., Yarza P., Peplies J., Glöckner F.O. (2013). The SILVA ribosomal RNA gene database project: Improved data processing and web-based tools. Nucleic Acids Res..

[41] Douglas G.M., Maffei V.J., Zaneveld J.R., Yurgel S.N., Brown J.R., Taylor C.M., Huttenhower C., Langille M.G.I. (2020). PICRUSt2 for prediction of metagenome functions. Nat. Biotechnol..

[42] McDonald D., Price M.N., Goodrich J., Nawrocki E.P., DeSantis T.Z., Probst A., Andersen G.L., Knight R., Hugenholtz P. (2012). An improved Greengenes taxonomy with explicit ranks for ecological and evolutionary analyses of bacteria and archaea. ISME J..

[43] Kanehisa M., Sato Y., Furumichi M., Morishima K., Tanabe M. (2019). New approach for understanding genome variations in KEGG. Nucleic Acids Res..

[44] Mangiola S., Doyle M.A., Papenfuss A.T. (2021). Interfacing Seurat with the R tidy universe. Bioinformatics.

[45] Oksanen J., Blanchet F.G., Kindt R., Legendre P., Minchin P., O’Hara R.B., Simpson G., Solymos P., Stevens M.H.H., Wagner H. (2013). Vegan: Community Ecology Package, R Package Version. 2.0-10.

[46] Min X., Wang Y., Chai L., Yang Z., Liao Q. (2017). High-resolution analyses reveal structural diversity patterns of microbial communities in Chromite Ore Processing Residue (COPR) contaminated soils. Chemosphere.

[47] Villanueva R.A.M., Chen Z.J. (2019). ggplot2: Elegant Graphics for Data Analysis.

[48] Hu K. (2021). Become Competent in Generating RNA-Seq Heat Maps in One Day for Novices Without Prior R Experience. Methods Mol. Biol..

[49] Slowikowski K., Schep A., Hughes S., Lukauskas S., Irisson J.O., Kamvar Z.N., Gramme P. ggrepel: Automatically Position Non-Overlapping Text Labels with ggplot2; 2018. https://CRAN.R-project.org/package=ggrepel.

[50] Aurand D., Coelho G. (2005). Cooperative Aquatic Toxicity Testing of Dispersed Oil and the “Chemical Response to Oil Spills: Ecological Effects Research Forum (CROSERF)” A Model for Cooperative Research by Industry and Government.

[51] Adams J.E., Bornstein J.M., Munno K., Hollebone B.P., King T.L., Brown R.S., Hodson P.V. (2014). Identification of compounds in heavy fuel oil that are chronically toxic to rainbow trout embryos by effects-driven chemical fractionation. Environ. Toxicol. Chem..

[52] Chand P., Dutta S., Mukherji S. (2022). Characterization and biodegradability assessment of water-soluble fraction of oily sludge using stir bar sorptive extraction and GCxGC-TOF MS. Environ. Pollut..

[53] Nelson R.K., Gosselin K.M., Hollander D.J., Murawski S.A., Gracia A., Reddy C.M., Radović J.R. (2019). Exploring the Complexity of Two Iconic Crude Oil Spills in the Gulf of Mexico (Ixtoc I and Deepwater Horizon) Using Comprehensive Two-Dimensional Gas Chromatography (GC × GC). Energy Fuels.

[54] Vulava V.M., Vaughn D.S., McKay L.D., Driese S.G., Cooper L.W., Menn F.M., Levine N.S., Sayler G.S. (2017). Flood-induced transport of PAHs from streambed coal tar deposits. Sci. Total Environ..

[55] Gao F., Zhou C., Wang Z., Zhu W., Wang X., Liu G. (2023). Solid-oil separation of coal tar residue to reduce polycyclic aromatic hydrocarbons via microwave-assisted extraction. J. Environ. Manag..

[56] Larsson M.O., Arp H.P.H., Carabante I., Kumpienė J. (2024). Evaluation and Modelling of Polycyclic Aromatic Hydrocarbon (PAH) Bioavailability in Soils Affected by Coal Tar Asphalt. Environ. Pollut..

[57] Lacina P., Mravcová L., Vávrová M. (2013). Application of comprehensive two-dimensional gas chromatography with mass spectrometric detection for the analysis of selected drug residues in wastewater and surface water. J. Environ. Sci..

[58] Skoczyńska E., Korytár P., Boer J.D. (2008). Maximizing Chromatographic Information from Environmental Extracts by GCxGC-ToF-MS. Environ. Sci. Technol..

[59] Flaks B. (1978). Effects of chronic oral dosing with quinine sulphate in the rat. Pathol. Res. Pract..

[60] Vuchetich P.J., Bagchi D., Bagchi M., Hassoun E.A., Tang L., Stohs S.J. (1996). Naphthalene-induced oxidative stress in rats and the protective effects of vitamin E succinate. Free Radic. Biol. Med..

[61] Bleeker E.A.J., Wiegman S., De Voogt P., Kraak M., Leslie H., Haas E., Admiraal W. (2002). Toxicity of azaarenes. Rev. Environ. Contam. Toxicol..

[62] Yuan X., Sun H., Guo D. (2012). The removal of COD from coking wastewater using extraction replacement–biodegradation coupling. Desalination.

[63] Wei X.X., Zhang Z.-Y., Fan Q.-L., Yuan X.-Y., Guo D.-S. (2012). The effect of treatment stages on the coking wastewater hazardous compounds and their toxicity. J. Hazard. Mater..

[64] Glushkov D.O., Paushkina K.K., Shabardin D.P. (2020). Co-combustion of coal processing waste, oil refining waste and municipal solid waste: Mechanism, characteristics, emissions. Chemosphere.

[65] Blum P., Sagner A., Tiehm A., Martus P., Wendel T., Grathwohl P. (2011). Importance of heterocylic aromatic compounds in monitored natural attenuation for coal tar contaminated aquifers: A review. J. Contam. Hydrol..

[66] Ordabaeva A.T., Muldakhmetov Z.M., Meiramov M.G., Kim S.V., Sagintaeva Z.I. (2025). Production of Pitch from Coal Tar of the Coke Chemical Production “Qarmet”. Molecules.

[67] Niksa S. (2017). A reaction mechanism for tar decomposition at moderate temperatures with any coal type. Fuel.

[68] Pereira P.H.F., Fernandes L., Jesus H.E., Costa P.G., Lacerda C.H.F., Mies M., Bianchini A., Santos H.F. (2023). The Impact of Highly Weathered Oil from the Most Extensive Oil Spill in Tropical Oceans (Brazil) on the Microbiome of the Coral Mussismilia harttii. Microorganisms.

[69] Schreiber L., Hunnie B., Altshuler I., Góngora E., Ellis M., Maynard C., Tremblay J., Wasserscheid J., Fortin N., Lee K. (2023). Long-term biodegradation of crude oil in high-arctic backshore sediments: The Baffin Island Oil Spill (BIOS) after nearly four decades. Environ. Res..

[70] Gutierrez T., Berry D., Teske A., Aitken M.D. (2016). Enrichment of Fusobacteria in Sea Surface Oil Slicks from the Deepwater Horizon Oil Spill. Microorganisms.

[71] Murphy S.M.C., Bautista M.A., Cramm M.A., Hubert C.R.J. (2021). Diesel and Crude Oil Biodegradation by Cold-Adapted Microbial Communities in the Labrador Sea. Appl. Environ. Microbiol..

[72] Wang C., Wu H., Zhao W., Zhu B., Yang J. (2024). Effects of Polycyclic Aromatic Hydrocarbons on Soil Bacterial and Fungal Communities in Soils. Diversity.

[73] Johnston E.L., Roberts D.A. (2009). Contaminants reduce the richness and evenness of marine communities: A review and meta-analysis. Environ. Pollut..

[74] Cai T., Ding Y., Zhang Z., Wang X., Wang T., Ren Y., Dong Y. (2019). Effects of total organic carbon content and leaching water volume on migration behavior of polycyclic aromatic hydrocarbons in soils by column leaching tests. Environ. Pollut..

[75] Shen X., Su X., Wan Y., Xu G., Lyu H., Song T., Dong W. (2024). Influence mechanisms of dissolved organic matter and iron minerals on naphthalene attenuation during river infiltration. Sci. Total Environ..

[76] Zhu Z., Merlin F., Yang M., Lee K., Chen B., Liu B., Cao Y., Song X., Ye X., Li Q.K. (2022). Recent advances in chemical and biological degradation of spilled oil: A review of dispersants application in the marine environment. J. Hazard. Mater..

[77] Brakstad O.G., Nordtug T., Throne-Holst M. (2015). Biodegradation of dispersed Macondo oil in seawater at low temperature and different oil droplet sizes. Mar. Pollut. Bull..

[78] Dubinsky E.A., Conrad M.E., Chakraborty R., Bill M., Borglin S.E., Hollibaugh J.T., Mason O.U., Piceno Y.M., Reid F.C., Stringfellow W.T. (2013). Succession of Hydrocarbon-Degrading Bacteria in the Aftermath of the Deepwater Horizon Oil Spill in the Gulf of Mexico. Environ. Sci. Technol..

[79] Ma Y., Wang L., Shao Z. (2006). Pseudomonas, the dominant polycyclic aromatic hydrocarbon-degrading bacteria isolated from Antarctic soils and the role of large plasmids in horizontal gene transfer. Environ. Microbiol..

[80] Wang M., Sha C., Wu J., Su J., Wu J., Wang Q., Tan J., Huang S. (2021). Bacterial community response to petroleum contamination in brackish tidal marsh sediments in the Yangtze River Estuary, China. J. Environ. Sci..

[81] Jiang S., Xue D., Feng W., Wang K., Wang S., Wang T., Lv M., Han Y., Lv Y., Hu A. (2024). Long-term organic fertilization alters soil microbial community structure and its influence on faba bean production in a six-crop rotation system. Plant Soil.

[82] Yang S., Xiao J., Liang T., He W., Tan H. (2021). Response of soil biological properties and bacterial diversity to different levels of nitrogen application in sugarcane fields. AMB Express.

[83] Lv J., Niu Y., Yuan R., Wang S. (2021). Different Responses of Bacterial and Archaeal Communities in River Sediments to Water Diversion and Seasonal Changes. Microorganisms.

[84] Li J., Zuo X., Chen Q., Lin Y., Meng F. (2025). Genome-resolved metagenomic analysis reveals a novel denitrifier with truncated nitrite reduction pathway from the genus SC-I-84. Water Res..

[85] Gorovtsov A.V., Sazykin I.S., Sazykina M.A. (2018). The influence of heavy metals, polyaromatic hydrocarbons, and polychlorinated biphenyls pollution on the development of antibiotic resistance in soils. Environ. Sci. Pollut. Res..

[86] Scariot M.A., Radünz L.L., Morelato R.R., da Costa Cabrera L., Dugatto J.S., Rohrig B., Dionello R.G., Radünz A.L. (2024). Contamination and persistence of polycyclic aromatic hydrocarbons (PAHs) in rice grains after drying in direct-fired dryer. Food Sci. Biotechnol..

[87] Thavamani P., Megharaj M., Naidu R. (2012). Bioremediation of high molecular weight polyaromatic hydrocarbons co-contaminated with metals in liquid and soil slurries by metal tolerant PAHs degrading bacterial consortium. Biodegradation.

[88] Sharma S., Singh K., Singh S. (2023). Synthetic Strategies for Quinoline Based Derivatives as Potential Bioactive Heterocycles. Curr. Org. Synth..

[89] Li Y., Li X., Cui Z., He F., Zong W., Liu R. (2023). Probing the toxic effect of quinoline to catalase and superoxide dismutase by multispectral method. Spectrochim. Acta A Mol. Biomol. Spectrosc..

[90] Mathe A., Mulpuru V., Katari S.K., Karlapudi A.P., Venkateswarulu T.C. (2024). Virtual screening and invitro evaluation of cyclooxygenase inhibitors from Tinospora cordifolia using the machine learning tool. J. Biomol. Struct. Dyn..

[91] Sun X., Zhuang J., Ma X., Tang Y., Ali M.M., Lu Z., Zheng X., Du Z. (2022). Structure elucidation and risk assessment of degradation products in gamma irradiated rubber closures. Polym. Degrad. Stab..

[92] Ma J., Ibekwe A.M., Crowley D.E., Yang C.-H. (2012). Persistence of Escherichia coli O157:H7 in Major Leafy Green Producing Soils. Environ. Sci. Technol..

[93] Capblancq T., Luu K., Blum M.G.B., Bazin E. (2018). Evaluation of redundancy analysis to identify signatures of local adaptation. Mol. Ecol. Resour..

[94] Pavía-Sanders A., Zhang S., Flores J.A., Sanders J.E., Raymond J.E., Wooley K.L. (2013). Robust Magnetic/Polymer Hybrid Nanoparticles Designed for Crude Oil Entrapment and Recovery in Aqueous Environments. ACS Nano.

[95] Borcard D., Gillet F., Legendre P. (2018). Numerical Ecology with R.

[96] Rogiers T., Claesen J., Van Gompel A., Vanhoudt N., Mysara M., Williamson A., Leys N., Van Houdt R., Boon N., Mijnendonckx K. (2021). Soil microbial community structure and functionality changes in response to long-term metal and radionuclide pollution. Environ. Microbiol..

[97] Dang C., Liu S., Chen Q., Sun W., Zhong H., Hu J., Liang E., Ni J. (2021). Response of microbial nitrogen transformation processes to antibiotic stress in a drinking water reservoir. Sci. Total Environ..

[98] Huang L., Ye J., Jiang K., Wang Y., Li Y. (2021). Oil contamination drives the transformation of soil microbial communities: Co-occurrence pattern, metabolic enzymes and culturable hydrocarbon-degrading bacteria. Ecotoxicol. Environ. Saf..

[99] Bacosa H.P., Erdner D.L., Rosenheim B.E., Shetty P., Seitz K.W., Baker B.J., Liu Z. (2018). Hydrocarbon degradation and response of seafloor sediment bacterial community in the northern Gulf of Mexico to light Louisiana sweet crude oil. ISME J..

[100] Zhou Y., Wang Y., Yang L., Kong Q., Zhang H. (2023). Microbial degradation mechanisms of surface petroleum contaminated seawater in a typical oil trading port. Environ. Pollut..

