# Supplementary material for: Different Emergency Response Strategies to Oil Spills in Rivers Lead to Divergent Contamination Compositions and Microbial Community Response Characteristics
Source: Microorganisms. 2025 May 23;13(6):1193. doi: 10.3390/microorganisms13061193 (PMC12195274; doi:10.3390/microorganisms13061193)
Supplement: Supplementary file 1 [file microorganisms-13-01193-s001.zip › microorganisms-3627112-supplementary.pdf]

Supplementary Information

Figure S1. Standard curve based on oil determination method (HJ 970 – 2018).

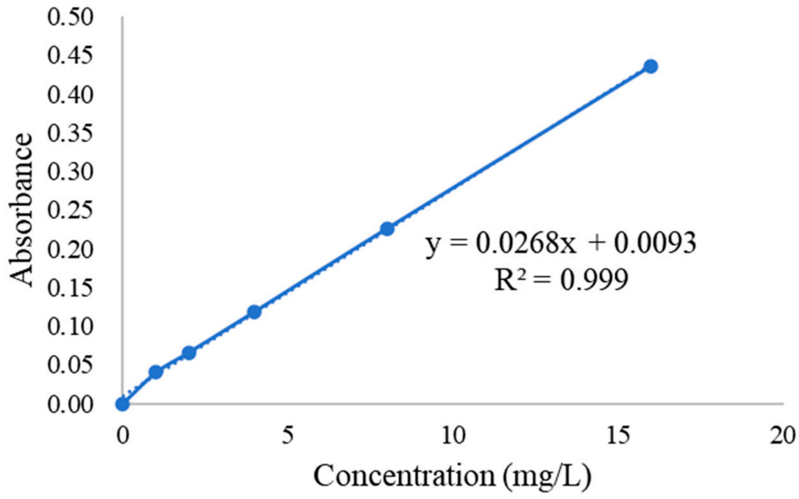

Table S1. The concentration of 10mg/L water containing washing oil was determined according to the method of HJ 970-2018, and the obtained results were all different from 10mg/L.

Sample Name	ϱ (mg/L)
10-1	0.250
10-2	0.238
10-3	0.242

Table S2. The absorbance of washing oil before and after water treatment was determined by HJ 970 – 2018. POL refers to the untreated contaminated sample.

Sample Name	pH	Absorbance
POL	6.06	3.798
ADT-1	6.20	3.360
ADT-2	6.18	3.484
ADT-3	6.27	3.484
AET-1	6.36	1.827
AET-2	6.22	1.913
AET-3	6.27	1.754
COT-1	4.47	3.405
COT-2	4.60	3.325
COT-3	4.67	3.325
DCT-1	9.51	1.087
DCT-2	9.56	0.706
DCT-3	9.53	0.945
